# Inhibiting microglia exacerbates the early effects of cuprizone in males in a rat model of multiple sclerosis, with no effect in females

**DOI:** 10.3389/fneur.2023.989132

**Published:** 2023-09-08

**Authors:** Soniya Xavier, Simin Younesi, Luba Sominsky, Sarah J. Spencer

**Affiliations:** ^1^School of Health and Biomedical Sciences, RMIT University, Melbourne, VIC, Australia; ^2^Barwon Health Laboratory, Barwon Health, University Hospital, Geelong, VIC, Australia; ^3^School of Medicine, Institute for Physical and Mental Health and Clinical Transformation, Deakin University, Geelong, VIC, Australia

**Keywords:** cuprizone, demyelination, females, microglia, multiple sclerosis

## Abstract

Hyper-activity of the brain’s innate immune cells, microglia, is a hallmark of multiple sclerosis (MS). However, it is not clear whether this involvement of microglia is beneficial or detrimental or whether manipulating microglial activity may be therapeutic. We investigated if inhibiting microglial activity with minocycline prevents the early changes in oligodendrocyte and myelin-related markers associated with a demyelinating challenge in adult female and male rats. Cuprizone reduced the expression of myelin and oligodendrocyte genes in both females and males, reflective of cuprizone intoxication and the early phases demyelination, and reduced the number of oligodendrocytes in the corpus callosum. However, we see notable differences in the role for microglia in this response between females and males. In males, myelin and oligodendrocyte genes, as well as oligodendrocytes were also reduced by minocycline treatment; an effect that was not seen in females. In males, but not females, early changes in oligodendrocyte and myelin-related genes were associated with microglial proliferation in corpus callosum, and this increase was reversed by minocycline. These data indicate sex-specific effects of inhibiting microglia on the early changes leading to demyelination in an MS model and suggest microglia may play a key role in myelin stability in males but not in females. This highlights a strong need for sex-specific understanding of disease development in MS and suggest that treatments targeting microglia may be more effective in males than in females due to differing mechanisms of disease progression.

## Introduction

Multiple sclerosis (MS) is one of the most common demyelinating diseases of the central nervous system (CNS). Both the symptoms and the severity of MS appear to be different between women and men, with women presenting with an earlier onset of the disease and having a higher rate of relapsing MS (1). While sex differences in the responses to genetic and environmental factors contribute to this disparity (2–4), the major sex-dependent contributors to this disease are unknown.

In addition to oligodendrocytes, myelin function is strongly supported by other glial cells, including microglia, the CNS’s innate immune cells (5). Microglia are present in MS lesion sites (6) and may be a key player in demyelination, since depleting microglia or blocking their activity prevents excessive demyelination in the corpus callosum in non-inflammatory pre-clinical MS models, at least in males (7–9). Notably, microglia show fundamental sex differences in their maturation process, distribution, and responses to challenges (10, 11). For instance, both the number and morphology of microglia are different in female and male mice during development (10). Current evidence suggests that differences in microglial kinetics may underlie different mechanisms behind the manifestation of chronic pain and of Alzheimer’s disease in women and men (11–13). We therefore hypothesized that the role of microglia in demyelination in MS differs depending upon sex.

In this study we induced an MS-like disruption to the myelination process in Wistar rats with the dietary supplement cuprizone. While a protective effect of removing microglia has previously been shown in females in an inflammatory MS model (14), cuprizone induces a pathophysiology and clinical signs in rodent models that closely resemble MS, without a substantial involvement of the peripheral immune cell infiltration into the CNS (15). It has been shown that the inflammatory responses during a demyelinating event are correlated with the myelin debris levels (16). Therefore, we examined the very early phases of cuprizone intoxication to assess the early involvement of microglia prior to their recruitment as phagocytes of cellular debris. Other groups have shown oligodendrocyte apoptosis occurs in the first few days after the commencement of the cuprizone diet. Oligodendrocytes and related genes are significantly altered at this time, while microgliosis and astrogliosis occur as the cuprizone diet is maintained, with degradation of the myelin sheath by about 3 weeks (17). To assess if inhibiting microglial activity could rescue these early disruptions to the myelination pathways in this model, we also gave the rats minocycline (or water) concomitant with the cuprizone to inhibit microglial activity (18, 19). In a notable clinical study, minocycline has been shown to lengthen the time between a first demyelinating event and the development of MS (20), making it a possible clinical treatment strategy, albeit that differences between genders was not investigated. Our findings here indicate there are significant differences in how microglia contribute to the development of MS in females and males.

## Materials and methods

### Animals

All experiments were conducted in accordance with the Australian Code of Practice for the Care and Use of Animals for Scientific Purposes, with approval from the RMIT University Animal Ethics Committee (AEC1803). In these experiments, we used 29 adult female and 28 adult male Wistar rats aged between 9 and 12 weeks obtained from our colony at the RMIT University Animal Facility. One animal was excluded due to more than 15% weight loss. The rats were allowed free access to water and standard rodent chow diet (Standard Rat and Mouse cubes; Specialty Feeds, Glen Forrest, WA, Australia) except where dietary manipulations were introduced, as described below.

### Cuprizone treatment

**Figure 1:**
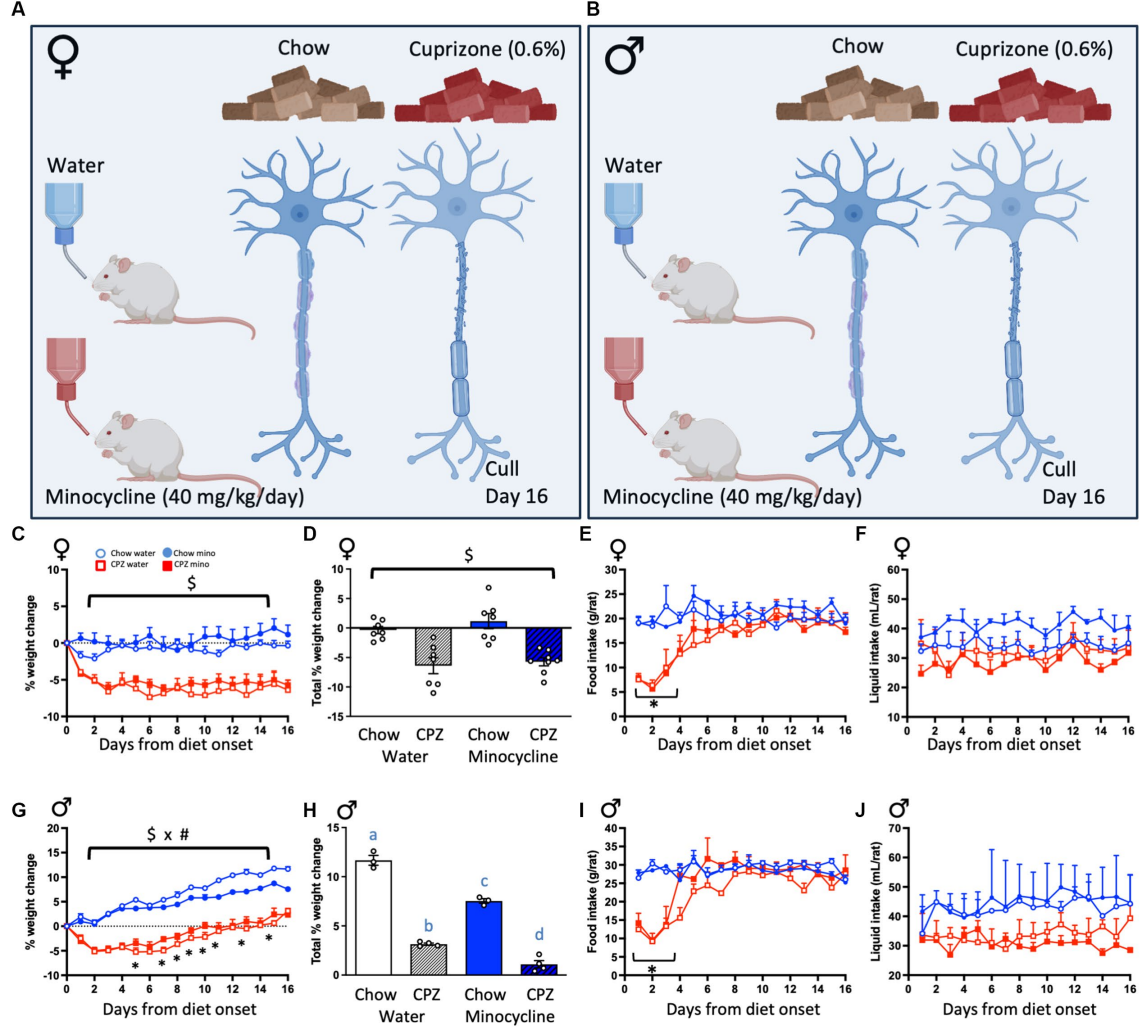
Experimental design and effects of cuprizone (CPZ) and minocycline on body weight, food and liquid consumption. **(A)** Female and **(B)** male rats were given either standard rat chow or a 0.6% cuprizone-supplemented diet *ad libitum* for 16 days and concomitantly treated with minocycline (equivalent to 40 mg/kg/day) in their drinking water, or normal tap water. All rats were culled 16 days after the start of the treatment protocol. Treatment groups were therefore chow-water, chow-minocycline, cuprizone-water or cuprizone-minocycline (females and males). In females: **(C)** Percentage weight change across the treatment period. **(D)** Percentage weight change between days 0 and 16. **(E)** Food intake. **(F)** Liquid intake. In males: **(G)** Percentage weight change. **(H)** Percentage weight change between days 0 and 16. **(I)** Food intake. **(J)** Liquid intake. Repeated measures analyses of variance (ANOVA) with time as the repeated measure, followed by one-way ANOVAs comparing diets at each day. **(C,D)**: $ denotes a significant main effect of cuprizone, **(E,I)**: * denotes a significant interaction between time and cuprizone with significant *post hoc* differences to the respective control groups. **(G)**: $ x # denotes a significant interaction between cuprizone and minocycline treatments. **(H)**: Different letters denote *post hoc* differences between groups. *p* < 0.05. Data are mean ± SEM. *n* = 5–8 per group. (A) and (B) created with BioRender.com; Toronto, Canada.

To induce cuprizone intoxication and disruption to oligodendrocytes and myelin-related genes, animals were provided with an *ad libitum* standard rat and mouse diet containing 0.6% cuprizone (SF18-032, Specialty Feeds) for 16 days (Figures 1A,B). Control rats continued to consume their standard rodent chow diet (Standard Rat and Mouse cubes; Specialty Feeds). The nutritional parameters of the control and cuprizone diets that we used in our study were identical except in the addition of 6 g/kg cuprizone (C9012, Sigma-Aldrich, Burlington, MA, United States) to SF18-032 (prepared by Specialty Feeds). Animals, food, and water bottles were weighed daily between 8 and 9 am to assess weight change and food and liquid consumption.

### Minocycline treatment

Concomitant with the consumption of cuprizone, rats were treated with either minocycline hydrochloride (equivalent to 40 mg/kg/day in drinking water; PCCA, Matraville, NSW, Australia) or minocycline-free tap water (Figures 1A,B). Minocycline was dissolved in drinking water, prepared fresh daily and provided *ad libitum*. Treatment groups therefore comprised female and male rats given either chow-water, chow-minocycline, cuprizone-water or cuprizone-minocycline (*n* = 5–8 per group).

### Tissue collection

At the end of the experiment, the rats were deeply anesthetized with Lethabarb (~150 mg/kg pentobarbitone sodium, i.p., Cenvet Australia Pty Ltd., Lynbrook, VIC, Australia) and brains extracted. All animals were culled between 8 and 10 am to minimize the impact of circadian rhythms on the outcomes. The left hemisphere was immersion-fixed in 4% paraformaldehyde in phosphate-buffered saline (PBS, pH 7.4) for 24 h and then placed into cryoprotectant with 20% sucrose in PBS (4°C). Fixed brains were sectioned coronally on a cryostat into a one in five series at 30 μm thickness and stored in 0.02% sodium azide at 4°C until used for immunohistochemistry. The corpus callosum was dissected from the right hemisphere, snap-frozen in liquid nitrogen and stored at −80°C until further processing.

### Gene expression

To assess changes in the expression of genes associated with inflammatory and myelinating processes, total RNA was extracted from the dissected corpus callosum using QIAzol reagents and RNeasy Mini Kits (Qiagen Valencia, CA, United States). RNA concentrations were determined as described previously (21). The comparative Ct method was used to analyse the data and target gene expression was normalized to the expression of the endogenous control gene *Gapdh*. Primer details are listed in Supplementary Table S1.

### Immunohistochemistry and analysis

#### Oligodendrocytes: Olig-2 and APC/CC-1

To assess the numbers of oligodendrocytes (mature and immature), sections through the corpus callosum were double-stained for oligodendrocyte transcription factor 2 (Olig-2) and adenomatous polyposis coli (APC; also known as APC clone (CC-1)). Olig-2 is a pan-nuclear marker present in all subtypes of the oligodendrocyte cell lineage. APC is a cytoplasmic marker specific for mature myelinating oligodendrocytes (22). Briefly, sections were washed with PBS and blocked for 1.5 h in 3% bovine serum albumin (BSA) and 0.3% Triton X-100 in 0.01 M PBS. Sections were then incubated with primary antibodies (rabbit polyclonal anti-Olig-2; 1:500, AB9610, Merck-Millipore, Burlington, MA, United States and mouse monoclonal anti-APC (CC-1); 1:500, AB16794, Abcam, Cambridge, England, United Kingdom) diluted in blocking buffer overnight at room temperature (RT). After washing, sections were incubated with secondary antibodies (Alexa-fluor 488 goat anti-rabbit; 1:250 and Alexa-fluor 594 donkey anti-mouse; 1:250, Thermo Fisher Scientific, Waltham, MA, United States) in 1% BSA, for 2 h at RT. After incubation, sections were washed to remove unbound secondary antibodies and then mounted using Dako Fluorescence mounting medium (Agilent, Santa Clara, CA, United States) and the slides stored at 4°C until imaging.

To quantify Olig-2 and APC positive cells, images from three sections through the corpus callosum between 2.76 and 3.48 mm caudal to the bregma were taken on a multiphoton microscope (A1 MP, Nikon Instruments Inc., Melville, NY, United States) at 20× magnification with Z-stacks of an average 6 steps with 5 μm between steps at a 1,024 × 1,024 resolution. A region of interest (ROI) within the corpus callosum was chosen from the acquired images and analysed using the “spots” function in Imaris software (Bitplane, Zurich, Switzerland). Each positive cell was detected by a spot and manually adjusted the threshold until all cells in the ROI were covered. A spot layer was individually created for each channel to detect Olig-2 and APC positive cells. The sum of the counts from all sections was used to obtain the total number of Olig-2 and APC positive oligodendrocytes.

#### Microglia: Iba1

To assess the numbers, density and morphology of microglia, forebrain coronal sections were immunolabelled for ionized calcium binding adaptor molecule 1 (Iba-1) as described previously (23). Briefly, sections were washed 3 × 10 min in 1 × PBS and blocked for 1 h with 3% BSA and 0.3% Triton X-100 in 1 X PBS. Sections were then incubated in primary antibody (rabbit polyclonal anti-Iba1, 1:1000, Wako Chemicals, Osaka, Japan) overnight, RT. Sections were washed again with 1 × PBS and incubated with the secondary antibody (1:500; Alexa-Fluor 488 goat anti-rabbit; Thermo Fisher Scientific) for 2 h at RT. Sections were then mounted onto Superfrost Plus slides and coverslipped with FluoroshieldTM with DAPI mounting medium (Sigma- Aldrich, St Louis, MO, United States).

To assess the number of microglia, the corpus callosum was located according to the Rat Brain Atlas of Paxinos and Watson (24). Images were taken on an upright confocal microscope (Nikon Eclipse 90i, Melville, NY, United States) using a 20x objective, with a 488 nm laser with a 515/30 filter set to detect fluorescently labelled Iba-1, and a 405 nm laser to detect DAPI. The numbers of Iba1-positive cells were determined manually by counting the cell soma using the cell counter tool in ImageJ (National Institute of Health, Bethesda, MD, United States). Four sections through the rostral corpus callosum, 120 μm apart, were analysed. The sum of the counts from all four sections was used to get the total number of microglia in our representative sample. As described previously (25), we assessed the density (coverage) of Iba-1-positive staining using the thresholding method in ImageJ (26). We also investigated microglial branching and process length by performing a skeletonization analysis using ImageJ (25). For this analysis, we converted the fluorescence images into an 8-bit grayscale image to best visualize all positive staining and then converted them to a binary image by adjusting the threshold. In addition, we also classified microglial morphology, as previously described (27, 28). Briefly, we categorised microglial cells as amoeboid (if there were zero to one primary processes), intermediate (if the cells had two to four primary processes) or ramified (if five or more primary processes were identified). All analysis was performed by an experimenter blinded to treatment conditions.

### Data analysis

All data were analysed using the Statistical Package for the Social Sciences for Windows (SPSS; IBM Corp., Armonk, NY, United States). To assess changes in body weight, food, and fluid intake, we used repeated measures analyses of variance (ANOVA) with day as the repeated measure. We used the Greenhouse–Geisser correction if the assumption of sphericity was violated and followed the analysis with two-way ANOVAs where significant interactions were found. For all other analysis we used two-way ANOVAs with cuprizone and minocycline as independent variables, followed by Tukey’s *post hoc* tests where significant interactions were found. Our primary aim was to describe the sex-specific effects of minocycline. Our primary case group was female rats, given the higher prevalence of MS in women than men. We therefore stratified the data by sex before presenting sex comparisons (multifactorial ANOVA). Full statistics on the sex comparisons are also reported in Supplementary Table S2. Data are presented as mean ± SEM and statistical significance was assumed when *p* ≤ 0.05.

## Results

### Effects of cuprizone and minocycline on body weight and food intake

To confirm sufficient ingestion of cuprizone and minocycline and to assess if these had any effect on basic metabolic parameters, we assessed body weight changes, as well as food and liquid intake throughout the treatment period. In females, cuprizone, in both the water and minocycline groups, reduced the percentage weight change from pre-treatment weights and minocycline had no effect either on the chow- or the cuprizone-fed groups (cuprizone effect: *F*_(1, 25)_ = 44.62, *p* < 0.001; Figure 1C). The percentage weight change between days 0 and 16 was also significantly reduced by cuprizone and not affected by minocycline (cuprizone effect: *F*_(1, 25)_ = 40.07, *p* < 0.001; Figure 1D). The female rats ate significantly less of the cuprizone-supplemented diet than the normal diet during the first 4 days of treatment (time x cuprizone interaction: *F*_(15, 120)_ = 7.31, *p* = 0.001 followed by one-way ANOVAs comparing diets at each day Figure 1E), after which time consumption of the cuprizone diet normalized. Neither treatment affected liquid consumption (Figure 1F).

In males, cuprizone, in both the water and minocycline groups, also significantly reduced weight across the experiment, with minocycline also reducing weight in the chow-fed animals in the last week of the study (time x cuprizone x minocycline interaction: *F*_(15, 165)_ = 2.88, *p* = 0.024; Figure 1G). The percentage weight change between days 0 and 16 was also significantly reduced by cuprizone and minocycline (cuprizone x minocycline interaction: *F*_(1, 10)_ = 10.35, *p* = 0.009; Figure 1H). As with the females, while the intake of cuprizone-supplemented diet was significantly reduced on days 1–4 of the treatment period (time x cuprizone interaction, *F*_(15, 90)_ = 11.10, *p* < 0.001; Figure 1I), food intake normalized to chow-fed control levels after day 4. As with females, there was no effect on liquid consumption (Figure 1J).

### Cuprizone and minocycline reduce the expression of oligodendrocyte genes in males but not females

**Figure 2:**
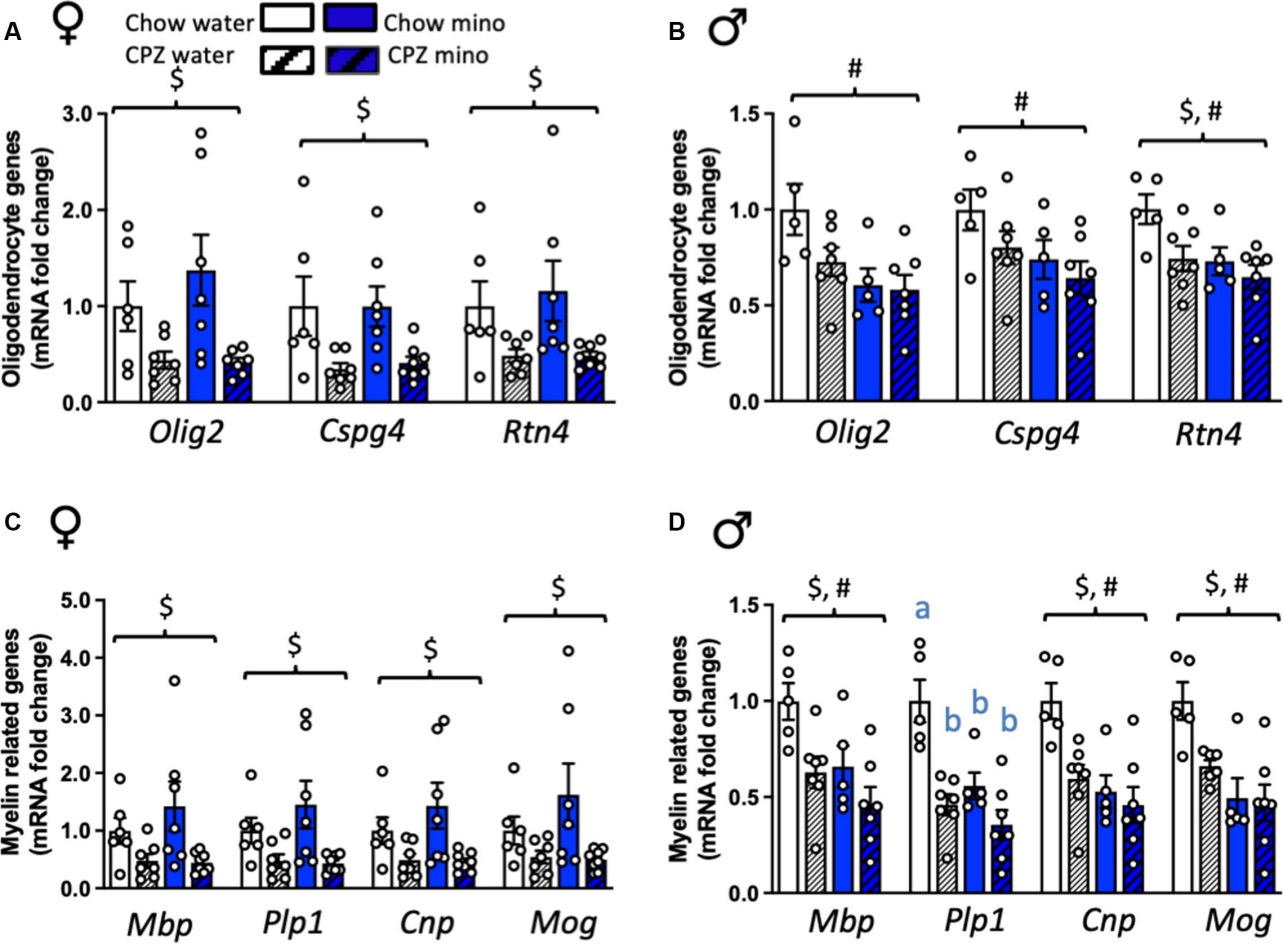
Effects of cuprizone (CPZ) and minocycline (mino) on the expression of oligodendrocyte and myelin-related genes in the corpus callosum. **(A)** Expression of oligodendrocyte-related genes, *Olig2, Cspg4*, and *Rtn4* in females. **(B)** Expression of *Olig2, Cspg4*, and *Rtn* in males. **(C)** Expression of myelin-related genes, *Mbp*, *Plp1, Cnp*, and *Mog* in females. **(D)** Expression of *Mbp*, *Plp1, Cnp*, and *Mog* in males. Two-way analysis of variance (ANOVA) with Tukey *post hoc*. $ denotes significant main effect of cuprizone, # denotes significant main effect of minocycline. Different letters denote *post hoc* differences between groups. *p* < 0.05. Data are mean ± SEM. *n* = 5–8 per group.

We next assessed the expression of oligodendrocyte- and myelin-related genes in the corpus callosum, a major myelinated fibre bundle that is affected in MS. In females, cuprizone significantly reduced the expression of all oligodendrocyte markers assessed, in both the water and minocycline groups, including *Olig2* and *Cspg4*, markers of oligodendrocyte progenitor cells (OPCs), and *Rtn4*, a marker for mature oligodendrocytes (*Olig2*: *F*_(1,23)_ = 11.08, *p* = 0.003; *Cspg4*: *F*_(1,24)_ = 12.55, *p* = 0.002 and *Rtn4*: *F*_(1,24)_ = 9.04, *p* = 0.006; Figure 2A). There was no effect of minocycline on any of oligodendrocyte-related genes we examined.

**Figure 3:**
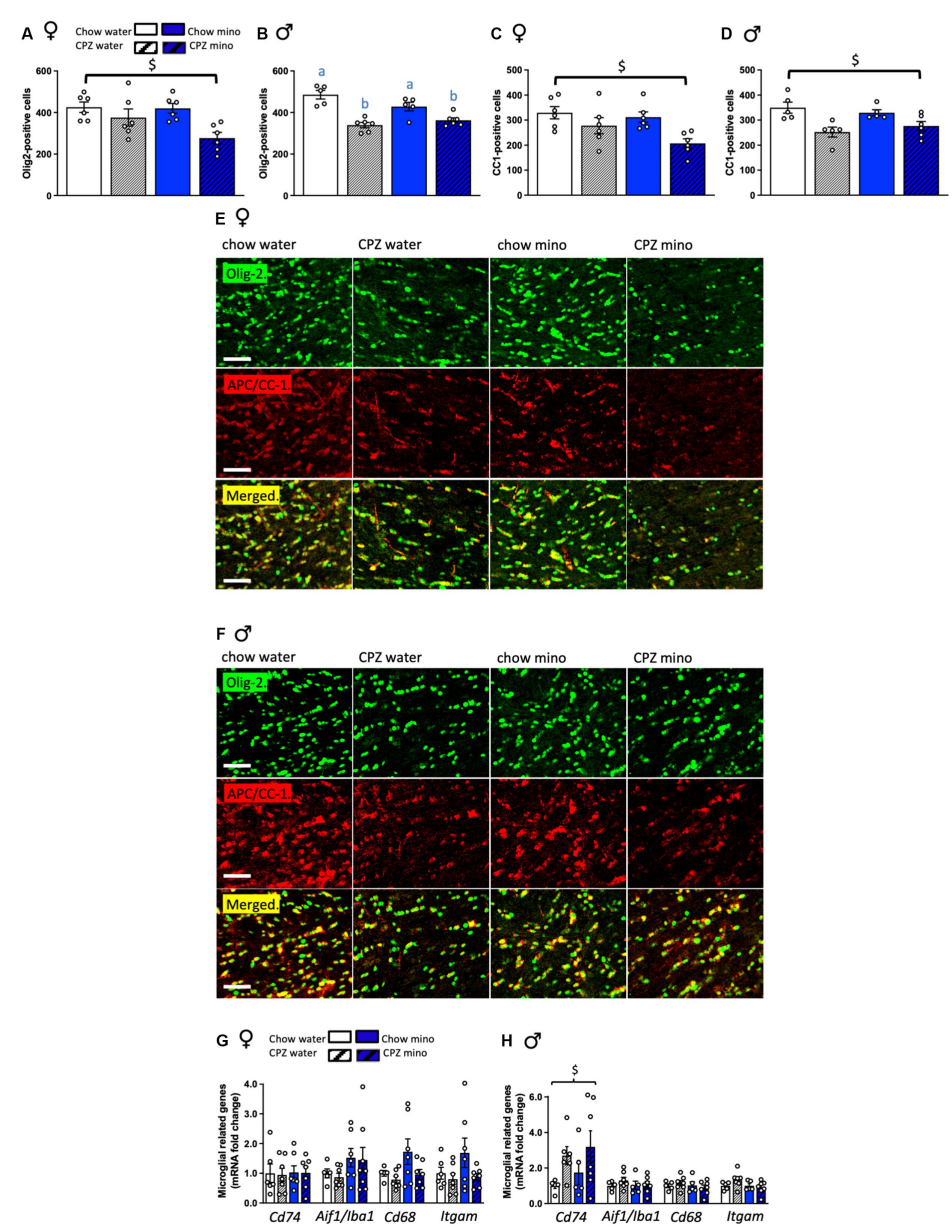
Effects of cuprizone (CPZ) and minocycline (mino) on Olig-2 and APC/CC-1 positive cells and the expression of microglia-related genes in the corpus callosum. **(A)** Number of Olig-2 positive cells in the corpus callosum of females. **(B)** Number of Olig-2 positive cells in the corpus callosum of males. **(C)** Number of APC/CC-1 positive mature oligodendrocytes in the corpus callosum of females. **(D)** Number of APC/CC-1 positive mature oligodendrocytes in the corpus callosum of males. **(E)** Representative photomicrographs of Olig-2 positive cells: upper panel, APC/CC-1 positive cells: middle panel and both Olig-2 and APC/CC-1 cells merged: bottom panel in the corpus callosum of females. **(F)** Representative photomicrographs of Olig-2 positive cells: upper panel, APC/CC-1 positive cells: middle panel and both Olig-2 and APC/CC-1 cells merged: bottom panel in the corpus callosum of males. **(G)** Expression of microglial-related genes, *Cd74, Aif1/Iba1, Cd68*, and *Itgam/Cd11b* in females. **(H)** Expression of microglial-related genes in males. Scale bar: 50 μm. Two-way analysis of variance (ANOVA) with Tukey *post hoc*. $ denotes significant main effect of cuprizone. Different letters denote *post hoc* differences between groups. *p* < 0.05. Data are mean ± SEM. *n* = 5–8 per group.

In males, however, *Olig2* and *Cspg4* were relatively protected against the cuprizone diet, showing no differences from chow-fed controls in expression at this time point (Figure 3B). *Rtn4* was significantly reduced by cuprizone in males (cuprizone effect: *F*_(1,20)_ = 5.83, *p* = 0.025). Surprisingly, though, minocycline significantly reduced the expression of all three of these genes in males (minocycline effect: *Olig2: F*_(1,20)_ = 8.57, *p* = 0.008; *Cspg4: F*_(1,20)_ = 4.79, *p* = 0.041; *Rtn4: F*_(1,20)_ = 6.86, *p* = 0.016; Figure 2B). There was a cuprizone by sex effect on *Olig2* and *Cspg4*, with a trend towards an interaction in *Rtn4*, indicative of sex differences in the response of these oligodendrocyte genes to cuprizone (*Olig2: F*_(1,43)_ = 5.56, *p* = 0.002; *Cspg4: F*_(1,43)_ = 5.59, *p* = 0.02; *Rtn4: F*_(1,43)_ = 3.64, *p* = 0.06; Supplementary Table S2).

### Cuprizone and minocycline reduce the expression of myelin genes in males but not females

In females, cuprizone, in both the water and minocycline groups, significantly reduced the expression of all myelin markers assessed (*Mbp*: *F*_(1,24)_ = 9.39, *p* = 0.005; *Plp1*: *F*_(1,24)_ = 10.68, *p* = 0.003; *Cnp*: *F*_(1,24)_ = 10.22, *p* = 0.004; and *Mog*: *F*_(1,24)_ = 6.98, *p* = 0.014; Figure 2C). Consistent with the oligodendrocyte markers, there was no effect of minocycline on the expression of any of these myelin-related genes. However, in males, cuprizone, in both the water and minocycline groups, and minocycline, in both the chow- and cuprizone-fed groups independently significantly reduced the expression of *Mbp* (cuprizone effect: *F*_(1,20)_ = 9.28, *p* = 0.006; main effect of minocycline: *F*_(1,20)_ = 7.41, *p* = 0.013), *Cnp* (cuprizone effect: *F*_(1,20)_ = 7.12, *p* = 0.015; main effect of minocycline: *F*_(1,20)_ = 11.99, *p* = 0.002) and *Mog* (cuprizone effect: *F*_(1,19)_ = 4.34, *p* = 0.05; main effect of minocycline: *F*_(1,19)_ = 15.60, *p* = 0.001; Figure 2D) with no interaction between the treatments. There was a significant interaction between cuprizone and minocycline on the expression of *Plp*, with a reduction in *Plp* expression in all the groups relative to the chow-water controls (cuprizone x minocycline interaction: *F*_(1,20)_ = 4.64, *p* = 0.044, Figure 2D). There was a minocycline by sex effect on *Cnp* and *Mog*, and *Plp1*, with a trend towards an interaction in *Mbp*, indicative of sex differences in the response of these myelin-related genes to minocycline (*Cnp: F*_(1,43)_ = 5.05, *p* = 0.03; *Mog: F*_(1,43)_ = 4.21, *p* = 0.05; *Plp1: F*_(1,43)_ = 4.22, *p* = 0.05; *Mbp: F*_(1,43)_ = 3.46, *p* = 0.07; Supplementary Table S2).

### Cuprizone and minocycline reduce the expression of mature and immature oligodendrocytes in males but not females

In females, cuprizone significantly reduced the number of Olig-2 positive cells in both the water and minocycline groups (*F*_(1,20)_ = 10.27, *p* = 0.005, Figures 3A,E). Consistent with the oligodendrocyte gene expression data, in females, there was no effect of minocycline on the Olig-2 positive cell numbers. In males, cuprizone significantly reduced the number of Olig-2 positive cells (main effect of cuprizone *F*_(1,18)_ = 42.46, *p* < 0.001, Figures 3B,F) and there was a significant interaction between cuprizone and minocycline treatment, with a reduction in Olig-2 numbers in minocycline treated cuprizone fed animals relative to the chow-water controls (cuprizone x minocycline interaction: *F*_(1,18)_ = 6.05, *p* = 0.024).

In both females and males cuprizone significantly reduced the number of mature APC/CC-1-positive cells (females: *F*_(1,20)_ = 10.19, *p* = 0.005; males: *F*_(1,17)_ = 16.82, *p* = 0.001, Figures 3C,D) with no effect of minocycline. There were no effects of sex on mature or immature oligodendrocyte numbers (Supplementary Table S2).

### Cuprizone increases the expression of microglial activation genes in males but not females

Since minocycline appeared ineffective in ameliorating cuprizone-related gene changes in females and was even detrimental in males, we next assessed expression of microglial genes in our groups. In females, neither cuprizone, in the water and minocycline groups, nor minocycline, in the chow- and cuprizone-fed groups altered corpus callosum microglial gene expression (Figure 3G). In males, however, cuprizone, in both the water and minocycline groups, significantly increased the expression of *Cd74* (*MhcII*; cuprizone effect: *F*_(1,19)_ = 5.09, *p* = 0.036, Figure 3H), potentially indicative of microglial pro-inflammatory activity. *Aif1* (*Iba1;* microglia marker), *Cd68* (monocyte marker), *Itgam* (*Cd11b*; leukocyte marker) were not significantly affected (Figure 3H). There was also a significant cuprizone by sex effect on the expression of *Cd74* indicative of sex differences in microglial response to cuprizone (*F*_(1,41)_ = 5.45, *p* = 0.025; Supplementary Table S2).

### Cuprizone increases microglial number and complexity in males but not females and this is ameliorated with minocycline

**Figure 4:**
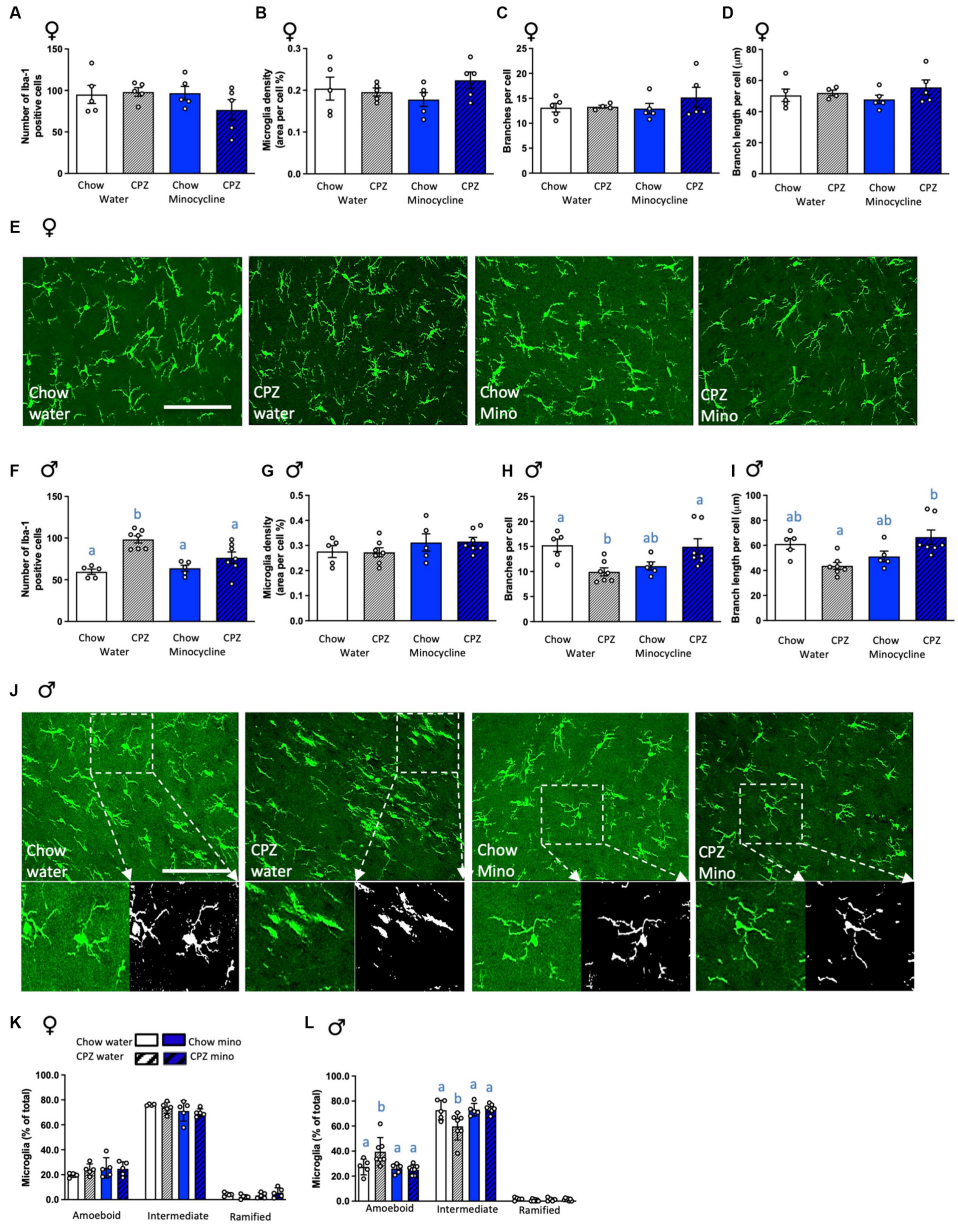
Effects of cuprizone and minocycline on microglial numbers and morphology in the corpus callosum. In females: **(A)** Number of Iba-1-positive cells in the corpus callosum. **(B)** Microglia density (coverage). **(C)** Number of branches per cell. **(D)** Branch length per cell. **(E)** Representative photomicrographs of Iba1-positive cells in the corpus callosum of females. In males: **(F)** Number of Iba-1-positive cells in the corpus callosum. **(G)** Microglia density (coverage). **(H)** Number of branches per cell. **(I)** Branch length per cell. **(J)** Representative photomicrographs of Iba1-positive cells in the corpus callosum of males in the upper panel with a larger magnification area showing the differences in microglia branch length and number of branches between treatment groups in the lower panel. The analysis output displaying branching and end points is in the lower right panel. Photomicrograph scale bars = 50 μm. CPZ: cuprizone; mino: minocycline. **(K)** Classification of microglial morphology in females. **(L)** Classification of microglial morphology in males. Two-way ANOVA with Tukey *post hocs*. Different letters denote *post hoc* differences between groups. *p* < 0.05. Data are mean ± SEM. *n* = 5–7 per group.

To assess if these changes in microglial gene expression were reflected in overt changes to microglial cells, we next assessed microglial numbers and morphology in our groups. In females, as for microglial gene expression, neither cuprizone, in the water and minocycline groups, nor minocycline, in the chow- and cuprizone-fed groups altered microglial numbers (Figure 4A) or density (Figure 4B). There were also no differences in microglial morphology in terms of the number of branches, process length per cell, or complexity classification (Figures 4C–E,K), together indicating that early changes to oligodendrocytes and myelin-related genes in females fail to activate a microglial response.

In males, as for microglial gene expression, cuprizone significantly increased the number of microglial cells in the corpus callosum in the water group and, notably, minocycline treatment ameliorated this effect (cuprizone x minocycline interaction: *F*_(1,20)_ = 6.26, *p* = 0.021; Figure 4F). Cuprizone and minocycline treatment did not affect the density of Iba-1 labelling (Figure 4G). However, the number of branches per cell was significantly reduced after cuprizone, indicative of reduced complexity, and again minocycline ameliorated this effect (cuprizone x minocycline interaction: *F*_(1,20)_ = 14.04, *p* = 0.001; Figure 4H). The branch length per cell was also significantly increased by minocycline after cuprizone (cuprizone x minocycline interaction: *F*_(1,20)_ = 13.13, *p* = 0.002, Figures 4I,J). These differences were further confirmed by the categorisation of microglial morphology into subcategories, with a significant increase in the percentage of amoeboid cells and a significant reduction in the percentage of those with intermediate morphology in cuprizone fed animals and minocycline treatment ameliorated this effect (amoeboid: cuprizone x minocycline interaction: *F*_(1,20)_ = 4.61, *p* = 0.044; intermediate: cuprizone x minocycline interaction: *F*_(1,20)_ = 4.81, *p* = 0.040; Figure 4L). Notably, there was a significant cuprizone by sex effect on microglial numbers (*F*_(1,35)_ = 11.08, *p* = 0.002), indicative of sex differences in the microglial response to early demyelination. There was also a significant minocycline by sex effect on numbers of ameboid (*F*_(1,35)_ = 7.23, *p* = 0.01) and intermediate (*F*_(1,35)_ = 8.17, *p* = 0.007; Supplementary Table S2) microglia, indicating minocycline affects microglial complexity differently in females and males.

## Discussion

In this study we aimed to assess if inhibition of microglial activity using minocycline could prevent the early disruption of the myelinating machinery in the corpus callosum of adult rats in a model of MS. As expected, two weeks’ consumption of cuprizone led to a reduction in the expression of myelin and oligodendrocyte-related genes in the corpus callosum of female and male rats, indicating early cuprizone-intoxication led to demyelinating events. In males, minocycline reduced the expression of both myelin and oligodendrocyte-related genes to the same extent as the cuprizone treatment, while there were no independent or additive effects of minocycline treatment on any myelin-related parameters in females. In males, but not females, early changes in oligodendrocyte- and myelin-related genes and oligodendrocyte numbers were associated with increased microglial numbers and reduced complexity, indicative of a pro-inflammatory microglial response, and minocycline ameliorated this effect. Our data thus suggest distinct differences in the response to challenges to the myelination process, and the role of microglia in these effects, in females and males. Since MS affects more women than men (29), it is essential to understand the molecular mechanism of demyelination using female animal models and our data indicate that the mechanisms are likely to be sex-dependent.

To date, very few studies have assessed the course of cellular and inflammatory responses associated with early demyelination in both sexes. In males, demyelination occurs when apoptosis of oligodendrocytes initiates the brain’s inflammatory cascade primarily by activating its immune cells, microglia, along with astrocytes (30). As the demyelination progresses, microglial activation also advances and the extent of the microglial response corresponds with the level of myelin debris (16). Therefore, in this study we investigated whether cuprizone-induced early microglial activation, prior to their recruitment as phagocytes of myelin debris. Since these processes have not been fully investigated in females, we assessed here whether they might occur differently in females and males. Our data suggest that the role of microglia in early demyelination may be beneficial in males, since suppressing microglial activity appeared to have an independent negative effect on myelination-related genes. It also appeared that in males, the expression of *Olig2* and *Cspg4*, markers of OPCs (22, 31), were relatively protected from cuprizone with no significant loss of expression. In females, on the other hand, cuprizone reduced the expression of *Olig2* and *Cspg4* along with *Rtn4,* a marker for mature oligodendrocytes, and manipulating the microglial contribution had no effect. A potential explanation for this difference in the expression of these genes could be that cuprizone toxin’s primary target is mature oligodendrocytes not OPCs (32), but that the effect on *Olig2* and *Cspg4* represents a more severe disease progression in the females. It is also noteworthy, that while the commencement of cuprizone treatment induced an acute reduction in food intake and weight loss in both sexes, cuprizone-treated females maintained a reduced body weight throughout the 16 days of cuprizone administration, while males appeared to begin to regain weight from day 5 onwards. However, the overall weight loss in cuprizone-treated rats remained to be significantly different from chow-fed rats throughout the experiment. Whether body weight in males is fully reinstated after a longer period of cuprizone exposure, whether this is different from females, and what are the effects of these metabolic differences on demyelination remains to be examined in future studies.

While the response of myelin-related gene expression to cuprizone was similar in both sexes, our data show a variability in microglial activation with sex. In males, but not in females, cuprizone significantly increased the expression of *Cd74* (MHCII), a marker of pro-inflammatory microglia (33). In line with the gene expression data, in males cuprizone led to more microglia that were less complex, while in females cuprizone did not affect microglial numbers or morphology, indicating sex differences in the contribution of microglia to the response of the myelination process to challenge. It is widely accepted that there is only a minor loss of blood–brain barrier (BBB) integrity and infiltration of peripheral immune cells, particularly lymphocytes, in cuprizone-induced metabolic oligodendrocyte degeneration and lesion development (34). However recent evidence suggests toxic demyelination is sufficient to trigger the recruitment of peripheral immune cells, mainly T cells, to the site of tissue injury when mice are exposed to cuprizone for 5 weeks. It does this without significant differences in the number of T cells at the first and third week of cuprizone treatment (35). Hence the microglial changes that we have seen during the two-week short-term cuprizone treatment are likely to be independent of any peripheral immune cell involvement. Although this has not been tested in rats, studies show that female mice are more resistant than males to cuprizone-induced loss of oligodendrocytes and demyelination without sex differences in microglial accumulation (36). Ovarian hormones have been shown to be involved in sex-specific microglial activation and it is therefore possible that in our study estrogen-induced neuroprotection prevented the early microglial response in females during the two-week cuprizone exposure.

Minocycline is commonly used to inhibit microglial activity and has been shown clinically to lengthen the time between the first demyelinating event and the development of MS in the first 6 months (20). While encouraging, how minocycline works to delay the risk of conversion of a clinically isolated syndrome to MS in these patients and whether this is sex dependent is unclear. Here, we show that although minocycline treatment did not ameliorate the cuprizone-induced increase in *Cd74* (MHCII) expression in male rats, it significantly reduced the cuprizone-associated increase in the total number of Iba1-positive cells. Minocycline treatment also alleviated cuprizone-induced morphological differences in the microglia, since no differences in morphology were evident in animals that had minocycline treatment along with cuprizone from control animals that had chow and water. Others have shown beneficial effects of minocycline treatment in inhibiting demyelination-associated microglial activation and therefore improving the myelination status of the corpus callosum (8–39). However, inhibiting microglial activation using minocycline during the re-myelination phase can hinder the myelination and differentiation of oligodendrocytes (40). Additionally, both cuprizone and minocycline can reduce the expression of myelin-related genes during demyelination (40) as we saw here in females. While there were no independent or additive effects of minocycline treatment on any parameters in females in our study, unexpectedly, minocycline reduced the expression of both myelin and oligodendrocyte-related genes to the same extent as the cuprizone treatment in males. Our findings indicate that minocycline can be as detrimental as cuprizone in orchestrating the cellular events that lead to demyelination, at least in males.

In conclusion, our study shows that microglial activation promotes early oligodendrocyte and myelin-related changes associated with cuprizone intoxication in the corpus callosum of female and male rats differently. These results indicate that minocycline may not be useful in preventing early demyelination in females and may even be detrimental in males. Our findings suggest further research is essential in both females and males to fully characterize the efficacy and safety of minocycline and other microglia inhibitors for use in MS.

## Data availability statement

The raw data supporting the conclusions of this article will be made available by the authors, without undue reservation.

## Ethics statement

The animal study was approved by RMIT University Animal Ethics Committee. The study was conducted in accordance with the local legislation and institutional requirements.

## Author contributions

SX, LS, and SS designed the study. SX and SY performed the experiments. SX, SY, LS, and SS contributed to analysis, writing, and editing the paper. All authors contributed to the article and approved the submitted version.

## Funding

This project was supported by funding from a National Health and Medical Research Council Career Development Fellowship II (APP1128646) to SS, Multiple Sclerosis Australia Incubator Grant to SS and LS, an RMIT Vice-Chancellor’s Postdoctoral Fellowship to LS, and RMIT University Ph.D. Scholarships to SX and SY. SS and LS are also supported by funding from an European Union (EU) Joint Program on Neurodegenerative Disease (JPND) Grant: (SOLID JPND2021-650-233). SS is further supported by an NHMRC Ideas Grant (2019196) and an Australian Research Council Discovery Project (ARC; DP230101331).

## Conflict of interest

The authors declare that the research was conducted in the absence of any commercial or financial relationships that could be construed as a potential conflict of interest.

## Publisher’s note

All claims expressed in this article are solely those of the authors and do not necessarily represent those of their affiliated organizations, or those of the publisher, the editors and the reviewers. Any product that may be evaluated in this article, or claim that may be made by its manufacturer, is not guaranteed or endorsed by the publisher.
