# Supplementary material for: Inhibiting microglia exacerbates the early effects of cuprizone in males in a rat model of multiple sclerosis, with no effect in females
Source: Front Neurol. 2023 Sep 8;14:989132. doi: 10.3389/fneur.2023.989132 (PMC10516553; doi:10.3389/fneur.2023.989132)
Supplement: Supplementary file 1 [file Data_Sheet_1.PDF]

RMIT Classification: Trusted

Supplementary Table 1. TaqMan probe details for qRT-PCR.

Target Gene	NCBI Reference Sequence	TaqMan Assay ID	Product Size
Gapdh	NM_017008.4	Rn01775763_g1	174
Aif1 (Iba1)	NM_017196.3	Rn00574125_g1	126
Cd11b (Itgam)	NM_012711.1	Rn00709342_m1	76
Cd68	NM_001031638.1	Rn01495634_g1	62
Cd74 (MhcII)	NM_013069.2	Rn00565062_m1	68
Cnp	NM_012809.2	Rn01399463_m1	100
Cspg4 (Ng2)	NM_031022.1	Rn00578849_m1	135
Gfap	NM_017009.2	Rn01253033_m1	75
Mbp	NM_001025291.1	Rn01399619_m1	80
Mog	NM_022668.2	Rn00575354_m1	62
Myrf	NM_001170487.1	Rn01454573_m1	81
Olig2	NM_001100557.1	Rn06344794_s1	83
Plp1	NM_030990.2	Rn01410490_m1	123
Rtn4 (NogoA)	NM_031831.1	Rn00582903_m1	90

Supplementary Table 2. Statistical comparisons by sex.

**Outcome measure**	**Cuprizone**	***p***	**Mino**	***p***	***Sex***	***p***	**Cuprizone x Mino**	***p***	**Cuprizone x Sex**	***p***	***Mino x Sex***	***p***
Cnp	$F_{(1,43)}$ = 13.08	<0.001	$F_{(1,43)}$ = 0.63	0.43	$F_{(1,43)}$ = 0.04	0.85	$F_{(1,43)}$ = 0.06	0.82	$F_{(1,43)}$ = 3.20	0.08	$F_{(1,43)}$ = 5.05	0.03
Mbp	$F_{(1,43)}$ = 13.14	0.001	$F_{(1,43)}$ = 0.24	0.63	$F_{(1,43)}$ = 0.01	0.98	$F_{(1,43)}$ = 0.33	0.57	$F_{(1,43)}$ = 2.49	0.12	$F_{(1,43)}$ = 3.46	0.07
Mog	$F_{(1,43)}$ = 8.56	0.005	$F_{(1,43)}$ = 0.16	0.69	$F_{(1,43)}$ = 0.34	0.56	$F_{(1,43)}$ = 0.15	0.70	$F_{(1,43)}$ = 2.64	0.11	$F_{(1,43)}$ = 4.21	0.05
Plp1	$F_{(1,43)}$ = 18.85	<0.001	$F_{(1,43)}$ = 0.34	0.56	$F_{(1,43)}$ = 0.44	0.51	$F_{(1,43)}$ = 0.06	0.82	$F_{(1,43)}$ = 1.53	0.22	$F_{(1,43)}$ = 4.22	0.05
Cspg4	$F_{(1,43)}$ = 14.06	0.001	$F_{(1,43)}$ = 0.92	0.34	$F_{(1,43)}$ = 1.31	0.26	$F_{(1,43)}$ = 0.07	0.79	$F_{(1,43)}$ = 5.59	0.02	$F_{(1,43)}$ = 1.01	0.32
Olig2	$F_{(1,43)}$ = 12.25	0.001	$F_{(1,43)}$ = 0.13	0.72	$F_{(1,43)}$ = 0.35	0.55	$F_{(1,43)}$ = 0.08	0.77	$F_{(1,43)}$ = 5.56	0.02	$F_{(1,43)}$ = 2.92	0.09
Rtn4	$F_{(1,43)}$ = 11.47	0.002	$F_{(1,43)}$ = 0.25	0.62	$F_{(1,43)}$ = 0.01	0.96	$F_{(1,43)}$ = 0.00	0.99	$F_{(1,43)}$ = 3.64	0.06	$F_{(1,43)}$ = 1.21	0.28
**Olig2 cells**	$F_{(1,37)}$ = 31.67	<0.001	$F_{(1,37)}$ = 3.57	0.07	$F_{(1,37)}$ = 2.60	0.11	$F_{(1,37)}$ = 0.02	0.88	$F_{(1,37)}$ = 0.09	0.76	$F_{(1,37)}$ = 0.98	0.33
**APC cells**	$F_{(1,37)}$ = 23.86	<0.001	$F_{(1,37)}$ = 1.81	0.19	$F_{(1,37)}$ = 1.67	0.20	$F_{(1,37)}$ = 0.02	0.89	$F_{(1,37)}$ = 0.01	0.92	$F_{(1,37)}$ = 2.13	0.15
Aif1	$F_{(1,41)}$ = 0.07	0.80	$F_{(1,41)}$ = 0.61	0.44	$F_{(1,41)}$ = 0.09	0.93	$F_{(1,41)}$ = 0.98	0.33	$F_{(1,41)}$ = 2.22	0.14	$F_{(1,41)}$ = 2.64	0.11
Cd11b	$F_{(1,41)}$ = 0.68	0.41	$F_{(1,41)}$ = 0.45	0.50	$F_{(1,41)}$ = 0.01	0.92	$F_{(1,41)}$ = 3.10	0.09	$F_{(1,41)}$ = 2.52	0.12	$F_{(1,41)}$ = 3.83	0.06
Cd68	$F_{(1,41)}$ = 2.13	0.15	$F_{(1,41)}$ = 1.00	0.32	$F_{(1,41)}$ = 0.36	0.55	$F_{(1,41)}$ = 1.88	0.18	$F_{(1,41)}$ = 2.58	0.12	$F_{(1,41)}$ = 3.46	0.07
Cd74	$F_{(1,41)}$ = 4.55	0.039	$F_{(1,41)}$ = 0.82	0.37	$F_{(1,41)}$ = 10.55	0.002	$F_{(1,41)}$ = 0.01	0.92	$F_{(1,41)}$ = 5.45	0.025	$F_{(1,41)}$ = 0.75	0.39

RMIT Classification: Trusted

**Iba1 cells**	$F_{(1,35)}$ = 2.26	0.14	$F_{(1,35)}$ = 2.79	0.10	$F_{(1,35)}$ = 9.84	0.004	$F_{(1,35)}$ = 4.91	0.03	$F_{(1,35)}$ = 11.08	0.002	$F_{(1,35)}$ = 0.00	0.99
**Density**	$F_{(1,35)}$ = 0.52	0.48	$F_{(1,35)}$ = 1.41	0.24	$F_{(1,35)}$ = 34.65	<0.001	$F_{(1,35)}$ = 0.76	0.39	$F_{(1,35)}$ = 0.49	0.49	$F_{(1,35)}$ = 1.78	0.19
**Branch/ cell**	$F_{(1,35)}$ = 0.08	0.78	$F_{(1,35)}$ = 0.51	0.48	$F_{(1,35)}$ = 0.89	0.35	$F_{(1,35)}$ = 9.81	0.003	$F_{(1,35)}$ = 1.17	0.29	$F_{(1,35)}$ = 0.05	0.83
**Length/ cell**	$F_{(1,35)}$ = 0.35	0.56	$F_{(1,35)}$ = 1.30	0.26	$F_{(1,35)}$ = 1.85	0.18	$F_{(1,35)}$ = 10.32	0.003	$F_{(1,35)}$ = 0.87	0.36	$F_{(1,35)}$ = 0.99	0.33
**Amoeboid**	$F_{(1,35)}$ = 3.20	0.08	$F_{(1,35)}$ = 1.27	0.27	$F_{(1,35)}$ = 8.57	0.006	$F_{(1,35)}$ = 4.82	0.03	$F_{(1,35)}$ = 0.87	0.36	$F_{(1,35)}$ = 7.23	0.01
**Intermediate**	$F_{(1,35)}$ = 3.74	0.06	$F_{(1,35)}$ = 0.48	0.50	$F_{(1,35)}$ = 1.80	0.19	$F_{(1,35)}$ = 3.19	0.08	$F_{(1,35)}$ = 0.99	0.33	$F_{(1,35)}$ = 8.17	0.007
**Ramified**	$F_{(1,35)}$ = 0.18	0.67	$F_{(1,35)}$ = 1.13	0.30	$F_{(1,35)}$ = 20.98	<0.001	$F_{(1,35)}$ = 5.31	0.03	$F_{(1,35)}$ = 0.48	0.49	$F_{(1,35)}$ = 1.03	0.32
